# Supplementary material for: Using Heatmap Visualization to assess the performance of the DJ30 and NASDAQ100 Indices under diverse VMA trading rules
Source: PLoS One. 2023 May 11;18(5):e0284918. doi: 10.1371/journal.pone.0284918 (PMC10174563; doi:10.1371/journal.pone.0284918)
Supplement: Attachment — Submitted filename: PONE-D-22-31748.pdf [file pone.0284918.s002.pdf]

PONE-D-22-31748: ***Using Heatmap Visualization to assess the performance of the DJ30 and NASDAQ100 Indices under diverse VMA trading rules***

The authors explore various VMA (variable-length moving average) trading rules and propose to use heatmap visualization to display the results. The authors use Dow Jones 30 and NASDAQ 100 as subjects of investigation and find greater return when using weekly data. The VMA trading rule is in general based on a long-term and a short-term moving average of an index, and once short-term MA is above (or below) long-term MA, it indicates a signal to buy (or sell) the index-linked product (see p.8 as well as Eq (2) & (3) in the manuscript). Although the Dow Jones and NASDAQ indexes are not really tradable, the authors would like to shed light on tracking the performance of exchange traded funds linked to these index as well as investment decision-making.

There are some major concerns relating to the interpretations and the contributions:

1 When the trading performance (i.e., Tables (2), (3), and onwards) is reported, it is pretty standard also to report the corresponding t-statistics for the averaged returns in empirical finance studies, because the viewers can decide whether the positive (or negative) return has statistical significance or just noise-driven returns (FN: since the test for statistical significance can be found in many classic statistics textbooks, please allow me not to provide reference). Particularly, in Table (3), the authors argue that the average return for weekly WMA (5, 120) is strikingly 60%, but the number of trades is just 9, whose occurrence is pretty scarce during the 2001–2020 sample period; I would be more careful about the interpretation and results.

   My concern over the lacking of statistical significance of course is also applied for the heatmap presentation. Again, the authors only present the averaged return in the heatmap, without considering the statistical tests. Then the information in heatmap is less reliable.

   There are two further suggestions on their trading performance in WMA:

   1.1 As said, Dow Jones and NASDAQ indexes are not really tradable, but transaction cost is pretty important to calculate performance. I wonder if the authors could use the bid / ask prices of the index constituents to approximate the transaction cost. Given the two indexes only contain 30 (or 100) stocks and index constituents are ready in Datastream, I would say calculating transaction is plausible.

1.2 Another suggestion is to add the stop-loss threshold for the WMA trading rules. Observing from some of the results, I find the positive return (let alone if they are statistically significant or not at the moment) often appears when there is a pretty long holding period (in some extreme cases, holding the portfolio nearly for two years). Since stop-loss is pretty common in terms of risk management, I wonder if the authors could investigate.

2 Finally, I feel somehow unconvinced how Heatmap Visualization could be stated as the contribution of the study. Indeed, with the aid of the heatmap, it's easier for the viewers to understand your whole results in a glance. However, since your heatmap is just static, there is limited information / usefulness of the heatmap. On the other hand, if you could provide a signaling method using *dynamic* heatmap and the heatmap-aided dynamic VMA rules is therefore beat the single / static VMA rules, then I would think a more appropriate contribution.

There are some minor suggestions:

1 It's not clear to me how you construct weekly WMA rules. Since the data is based on daily frequency, do you enter the trade on Mondays only and exit on Fridays only?

2 Inappropriate citations:

"As a result of inflationary concerns, many people may be motivated to invest in stock markets rather than banks, attempting to understand the stock market, predict stock prices, generate satisfactory profits, and become successful investors" (p. 3) and you cited Roundy et al. (2017). I quickly read the paper and I don't think this citation is relevant to your statement here, as Roundy et al. (2017) is to provide difference between impact investment and social responsibility investment, I don't see how this is related to inflation-driven investment.

Another example of inappropriate citation is "according to the efficient market hypothesis (EMH), shock (PS, this should be a typo, it should be *stock*) prices have reflected all available information" (p. 3), and you cite several studies by Fama. It's true that Fama pioneers the conceptualization of efficient financial markets, but it's well known that there

are different levels of efficient markets, and a financial market can be still efficient even some information is not yet reflected. So the statement here could be misleading.

I would like to thank the authors for giving me the opportunity to review your paper. It's an interesting one but I would think the credibility of the results require much further investigation.

Reference:

[1] Roundy, P., Holzhauer, H. and Dai, Y., 2017. Finance or philanthropy? Exploring the motivations and criteria of impact investors. *Social Responsibility Journal.*

[2] Fama, E.F., 1970. Efficient capital markets: A review of theory and empirical work. *Journal of Finance*, 25(2), pp.383-417.

[3] Fama, E.F., 1991. Efficient capital markets: II. *Journal of finance*, 46(5), pp.1575-1617
