# Supplementary material for: Using Heatmap Visualization to assess the performance of the DJ30 and NASDAQ100 Indices under diverse VMA trading rules
Source: PLoS One. 2023 May 11;18(5):e0284918. doi: 10.1371/journal.pone.0284918 (PMC10174563; doi:10.1371/journal.pone.0284918)
Supplement: Attachment — Submitted filename: Response to Reviewers.docx [file pone.0284918.s003.docx]

**Dear Editor and Reviewers,**

We are glad and grateful for the opportunity to improve our manuscript’s quality. We much appreciate your constructive comments, intellectually stimulating suggestions, and encouraging words. We have carefully considered all the comments and revised our manuscript accordingly and the changes and revisions made in this manuscript are marked in RED. Thank you for providing valuable and insightful comments, which indeed help us to learn how to write a top-tier academic paper and how to enhance our manuscript. Thank you so much.

**REVIEWER 1**

| **Suggestions/comments from the Reviewer** | **Response from the Author(s)** |
| --- | --- |
| Please find the attached file in my attachments to see my detailed comments. In this report, I would like to point some points which might be helpful for the authors to improve the quality of the manuscript before it gets published.  **Summary**  This study explores whether using various VMA trading rules would improve investment performance due to the flexibility of VMA trading rules and the aid of heat-map visualization. The results of the empirical analysis show that the NASDAQ100 index outperforms the DJ30 index and that weekly data outperforms daily data when measured by annualized return. In addition, it provided several insights for policymakers and investors in the stock market. Generally speaking, the writing of the manuscript reflects the professionalism of the authors. But before it can be accepted for publication, the authors need to make the following major revisions to the existing manuscript. | Thank you for your valuable feedback on our study. We greatly appreciate the time and effort you have taken to review our manuscript. Your comments and suggestions will be very helpful in improving this manuscript. We are committed to making the necessary revisions to our manuscript and addressing the points you have raised. Your insights play a critical role in helping us to enhance this paper. Thank you so much. |
| **Comments:** |  |
| 1. The authors mentioned in the introduction part that “Profitability trading using technical trading rules appears to be welcomed by market participants and even academic researchers, which would be the research background and motivation for this study.” It is suggested that the authors restate the motivation in this paper. It could be an unsolved problem from previous studies or a practical problem in the rules of market trading (relevant to the theme of this paper). | Thank you for your feedback and suggestions on our paper. Indeed, we should enhance the motivation of this study. In light of your suggestion, we have restated the motivation in this paper. Thank you so much. **(please see P. 4)** |
| 2. The fourth point of the study's contribution is not obvious. It is suggested that the authors connect the fourth contribution with the third point (relevant to the heat-map visualization). | Thank you for your treasured comment and suggestions. We totally agree with your valuable comment that connecting the fourth contribution with the third point would provide a clearer understanding of our study's contributions. Thank you very much. **(please see P. 7)** |
| 3. Some literature about the implications as well as techniques need to be updated carefully:   [a] Leviton, L. C., & Trujillo, M. D. (2017). Interaction of theory and practice to assess external validity. *Evaluation Review*, 41(5), 436-471.  [b] Kwok, J.K.H. (2020), "Does switching trading venues create value? Evidence from Hong Kong", *Journal of Asian Business and Economic Studies*, Vol. 27 No. 2, pp. 209-222. https://doi.org/10.1108/JABES-09-2019-0080 | Thank you for pointing out the need to update the literature in the study's implications and techniques. We incorporate these references [a] Leviton and Trujillo (2017) and [b] Kwok (2020) in the context of this manuscript. Many thanks. **(please see P. 11, P. 21, and P. 27)** |
| 4. It is recommended that the authors add robustness tests following the main empirical work to support the results of the study. For example, the authors mentioned that the DJ30 and NASDAQ 100 indices were chosen because these stock indices enjoy high attention from investors and have high trading volume. Can other similar stock indices be used for comparison to illustrate the effectiveness of the VMA trading rules? | According to your insightful suggestion, we then utilize the S&P 500 index, which may be regarded as the most representative index for US stock markets (Yousfi et al., 2021), the representative of global stock markets (Nandy & Chattopadhyay, 2019), and even the primary representation of the global economy (Verma et al., 2021), because the S&P 500 is a stock market index composed of the 500 largest US companies by market capitalization. Following that, the results obtained by using the S&P 500 index are comparable to those obtained by using the DJ 30 and NASDAQ indices. We conclude that similar outcomes are because these indices are US stock indices. To save space, we then present and explain our results as shown below instead of increasing more space in the context. Thank you so much. **(Please see the empirical results using the S&P 500 index below.)** |
| **Empirical results using the S&P 500 index** ***Empirical results of the traditional design*** Because 5-day and 20-day moving averages are considered weekly and monthly moving averages in practice, we employ VMA trading rules such as VMA (5, 20*N), where N=1-6, which is set as the traditional design. Because institutional investors recommend long-term investments, we use weekly data instead of daily data, and the results are shown in Table A. **Table A** VMA Trading Strategies for the S&P 500 index from 2001 to 2020 (1) \| (2) \| \| (3) \| \| (4) \| \| (5) \| \| (6) \|; Strategy \| No. of Trades \| \| Total Return (%) \| \| GM (%) \| \| Avg. Duration Day \| \| Max. Duration Day \|; (5, 20) \| \| 56 \| \| 31.17 \| \| 0.56 \| \| 18 \| \| 93; (5, 40) \| \| 29 \| \| 63.93 \| \| 2.20 \| \| 34 \| \| 188; (5, 60) \| \| 19 \| \| 30.96 \| \| 1.63 \| \| 48 \| \| 189; (5, 80) \| \| 15 \| \| 21.98 \| \| 1.47 \| \| 61 \| \| 239; (5, 100) \| \| 13 \| \| 35.57 \| \| 2.74 \| \| 70 \| \| 234; (5, 120) \| \| 13 \| \| 143.93 \| \| 11.10 \| \| 69 \| \| 228 Table A shows that the S&P 500 index performance using the VMA (5, 120) trading rule has the highest GM (11.07%), which is higher than the GMs using other VMA trading rules in Table A. However, investors would be interested in obtaining much higher GMs by employing more VMA trading rules. Thus, we investigate whether different SMA and LMA combinations in a heatmap matrix yield higher GMs than the highest GM in the preceding design. ***Empirical results from a heatmap matrix*** Our design is to generate the higher GMs among numerous GMs by employing numerous VMA trading rules, as shown in Table B (i.e., the results of VMA (N1, N2)) shown in a 6-by-23 heatmap matrix or Figure A (i.e., with cells ranging from dark blue (representing much lower GMs) to bright yellow (representing much higher GMs)). Following that, we would find red color cells representing higher ARs among numerous GMs in Table A or bright color cells representing higher GMs via heatmap visualization in Figure A. Furthermore, the results in red cells (over 11%) in Table B are higher than the highest return (i.e., 11.1%) in Table A. We thus argue that our design is worthwhile for investors trading S&P500 ETFs. **Table B.** The heatmap matrix using diverse VMA trading rules Unit: % In Table 3, N1is from 5 weeks to 30 weeks, N2 is from 10 weeks to 120 weeks, N2>N1 (i.e., in blue), and the time interval is 5 weeks; the cells in red indicate that GMs are all over 11.1% (i.e., the highest GM in Table 2). **120** \| **11.1** \| **16.7** \| **20.0** \| **35.6** \| **25.2** \| **38.8**; **115** \| **2.1** \| **11.3** \| **21.4** \| **24.4** \| **36.6** \| **22.6**; **110** \| **2.0** \| **12.1** \| **19.7** \| **25.5** \| **23.0** \| **21.9**; **105** \| **2.6** \| **9.6** \| **13.3** \| **22.9** \| **30.9** \| **32.0**; **100** \| **2.7** \| **8.5** \| **13.3** \| **14.3** \| **31.5** \| **32.9**; **95** \| **1.9** \| **7.2** \| **11.3** \| **14.5** \| **20.1** \| **31.9**; **90** \| **2.4** \| **5.4** \| **9.6** \| **12.5** \| **19.5** \| **29.9**; **85** \| **2.1** \| **7.0** \| **8.4** \| **9.2** \| **11.5** \| **19.3**; **80** \| **1.5** \| **6.1** \| **8.2** \| **9.7** \| **11.9** \| **14.2**; **75** \| **1.7** \| **5.5** \| **9.2** \| **10.1** \| **9.5** \| **10.0**; **70** \| **2.4** \| **6.0** \| **5.8** \| **10.8** \| **10.7** \| **11.3**; **65** \| **1.3** \| **5.7** \| **5.3** \| **10.3** \| **10.6** \| **10.3**; **60** \| **1.6** \| **4.8** \| **2.8** \| **5.1** \| **7.8** \| **10.1**; **55** \| **0.2** \| **4.3** \| **1.5** \| **4.9** \| **8.7** \| **8.5**; **50** \| **2.3** \| **3.7** \| **3.0** \| **3.4** \| **4.3** \| **6.6**; **45** \| **3.2** \| **2.9** \| **3.1** \| **1.9** \| **5.2** \| **1.9**; **40** \| **2.2** \| **2.1** \| **1.9** \| **1.6** \| **2.8** \| **3.5**; **35** \| **2.8** \| **2.7** \| **0.7** \| **1.3** \| **1.5** \| **1.7**; **30** \| **0.8** \| **2.1** \| **0.2** \| **0.4** \| **0.8** \|; **25** \| **-0.6** \| **0.6** \| **-0.7** \| **0.3** \| \|; **20** \| **-0.6** \| **0.3** \| **-0.7** \| \| \|; **15** \| **0.4** \| **0.3** \| \| \| \|; **10** \| **-0.1** \| \| \| \| \|; **N2 / N1** \| **5** \| **10** \| **15** \| **20** \| **25** \| **30**  **Figure A.** Heatmap Visualization of trading results based on various VMA trading rules. | |
| 5. It is suggested that the authors remove the statement to the contribution of this study from the conclusion from page 19 to page 20. The current version of the conclusion is too redundant. This would give the reader the impression that the results of this study is weak to support the conclusions. | Thank you for your valuable feedback. We agree that the conclusion in the previous version is too redundant, giving the reader the impression that the results of this study are weak to support the conclusions. As a result, we removed the redundant statement from the conclusion section as suggested.  Once again, thank you for your constructive feedback and suggestions. **(Please see the concluding remark section; P. 20- P. 23)** |
| 6. The existing reference may have some problems with the format. For instance, the authors mentioned “According to Brock et al. [21] (1992)” on **page 4.** It is suggested that the authors double-check the existing format of reference and revise them carefully in the whole text. | Thank you for your treasured comment. We would double-check the format of our references in the context. In other words, we will carefully review and revise the reference format throughout the text to ensure that it meets the Journal standards. Thank you so much. **(Please see P. 4)** |
| 7. It is recommended that the authors recheck the formulas and their interpretation in the research design. The current version seems to be not in good order (e.g. position of formula serial numbers and line spacing). | Thank you for your valuable comment. Because the formula serial numbers and line spacing were not presented professionally in the previous version, we rechecked and revised the formula presentation and interpretation. **(Please see P. 8 – P. 11)** |
| We greatly appreciate your effort in reviewing this paper and your treasured comments provided for our paper, all of which would indeed help us to learn how to write a good academic paper and how to enhance our manuscript. Although we have to make efforts to revise this paper, we indeed learn a lot. We are really appreciated your valuable comments and suggestions. Thank you so much. | |

**REVIEWER 2**

| **Suggestions/comments from the Reviewer** | **Response from the Author(s)** |
| --- | --- |
| The authors explore various VMA (variable-length moving average) trading rules and propose to use heatmap visualization to display the results. The authors use Dow Jones 30 and NASDAQ 100 as subjects of investigation and find greater return when using weekly data. The VMA trading rule is in general based on a long-term and a short-term moving average of an index, and once short-term MA is above (or below) long-term MA, it indicates a signal to buy (or sell) the index-linked product (see p.8 as well as Eq (2) & (3) in the manuscript). Although the Dow Jones and NASDAQ indexes are not really tradable, the authors would like to shed light on tracking the performance of exchange traded funds linked to these index as well as investment decision-making. | Thank you for taking the time to review our manuscript. We appreciate your constructive feedback and are glad to see that you have a clear understanding of our proposed VMA trading rules and heatmap visualization. We agree that the Dow Jones 30 and NASDAQ 100 indices are not directly tradable, but our goal was to demonstrate the application of the VMA rules and heatmap visualization to track the performance of the ETFs linked to these indexes for investment decision-making purposes. Thank you again for your insightful comments. |
| **Comments:** |  |
| 1. When the trading performance (i.e., Tables (2), (3), and onwards) is reported, it is pretty standard also to report the corresponding t-statistics for the averaged returns in empirical finance studies, because the viewers can decide whether the positive (or negative) return has statistical significance or just noise-driven returns (FN: since the test for statistical significance can be found in many classic statistics textbooks, please allow me not to provide reference). Particularly, in Table (3), the authors argue that the average return for weekly WMA (5, 120) is strikingly 60%, but the number of trades is just 9, whose occurrence is pretty scarce during the 2001–2020 sample period; I would be more careful about the interpretation and results.  My concern over the lacking of statistical significance of course is also applied for the heatmap presentation. Again, the authors only present the averaged return in the heatmap, without considering the statistical tests. Then the information in heatmap is less reliable.  There are two further suggestions on their trading performance in WMA: | Thank you for your valuable comment. Based on your comment “the average return for weekly VMA (5, 120) is strikingly 60%, but the number of trades is just 9, whose occurrence is pretty scarce during the 2001–2020 sample period", we totally agree with you that the sample size is limited even though we use a long-period data (i.e., 20-year data). As a result, we would be more cautious about the interpretation and results. Furthermore, we would point out this fact as a limitation of this study, since limited trading signals generated by several VMA trading rules would be a limitation of this study. Thank you so much. (Please see P. 23 – P. 24)  Even though we used 20-year data, the sample size for several VMA trading rules would be limited due to the study's use of weekly data. Furthermore, presenting so many performance results (GAs) in two heatmap matrices may help the audience understand the results of numerous VMA trading rules.  However, presenting detailed information (such as sample size, t value, and P value for each of numerous VMA trading rules) in each cell of a heatmap matrix properly might not be an easy task as well.  As previously stated, because we used weekly data, the sample size for several VMA trading rules would be limited, even though we put the above information (including sample size, t value, and P value) in two heatmap matrices. In other words, the sample size for multiple VMA trading rules would be still less than 30.  Nonetheless, despite the limited sample size, the many GAs shown in two heatmap matrices that are calculated using secondary historical data would be true.  We completely agree with your valuable comment, but the issues raised by your valuable comment may be difficult to resolve in a short period of time unless a large and diverse data set is used. Based on your feedback, we believe that we should conduct additional research using the constituent stocks of the DJ 30 and NDX 100 indices, which should provide enough samples for diverse VMA trading rules and provide more reliable results in our future studies. As a result, we would consider your treasure concern in this study by incorporating your valuable concern into the limitations and future research of this study. (Please see P. 23- P. 24) |
| 1.1 As said, Dow Jones and NASDAQ indexes are not really tradable, but transaction cost is pretty important to calculate performance. I wonder if the authors could use the bid / ask prices of the index constituents to approximate the transaction cost. Given the two indexes only contain 30 (or 100) stocks and index constituents are ready in Datastream, I would say calculating transaction is plausible. | Thank you for your thoughtful comment and suggestions.  What we are concerned about is that, given the widespread ownership of DIA and QQQ, two of the most popular ETFs that track the DJ 30 and NASDAQ 100 indexes, by many investors, we use these two indices with long-term periods as our samples. Furthermore, to our understanding, the transaction cost of ETFs is lower than the cost of trading the constituent stocks of these two indices.  In addition, because our revealed results are derived from these two indices, calculating the transaction cost for DIA and QQE may be more appropriate because the performance of DIA and QQQ tracks those of these two indices. Furthermore, the GAs shown in the heatmap are rather impressive for investors, so the GA performance would be still impressive after subtracting the transaction cost, as the transaction cost of these two ETFs would be minimal.  We agree that investors can trade the constituent stocks of these two indices; however, different brokers will charge different transaction costs, and transaction costs may vary depending on the trading volume of these constituent stocks. Based on the foregoing, determining the objective transaction costs for more than 100 stocks (i.e., 130 stocks) in a short time may not be easy for researchers in non-US countries rather than US institutional traders.  As previously stated, if we use the constituent stocks of these two indices as our sample in future studies, using the transaction cost would be more compatible and plausible. Furthermore, if the revealed results (GAs) are still impressive as disclosed in this study, the revealed results would be still impressive after subtracting transaction costs, due to the competition of a variety of brokers (broker enterprises) in the broker industry. |
| 1.2 Another suggestion is to add the stop-loss threshold for the VMA trading rules. Observing from some of the results, I find the positive return (let alone if they are statistically significant or not at the moment) often appears when there is a pretty long holding period (in some extreme cases, holding the portfolio nearly for two years). Since stop-loss is pretty common in terms of risk management, I wonder if the authors could investigate. | Thank you for your insightful comments, which enabled me to think more deliberately about this research. Thank you incredibly much. Indeed, according to VMA trading rules, this transaction included a stop-loss mechanism (i.e., exit as dead-cross emitted). That is, using the VMA trading rule, there is an obvious rule for existing or stop-loss. However, there is no universal stop-loss threshold, and different thresholds may produce inconsistent results.  Furthermore, while observing the trend of these two indices, using stop-loss mechanisms may reduce loss in the short run. However, as shown in Figure 1, investors using a stop-loss strategy may occur in a sharp drop in the trend. However, we argue that if investors buy instead of stop loss the EFTs tracking these two indices at the sharp drop in the trend, they may reap profit, even satisfactory profits, in the long run. In addition, according to Warren Buffett, holding index ETFs such as S&P 500 ETFs would provide satisfactory long-term performance because his suggestion (holding S&P 500 ETF) outperformed most active mutual funds, which are also publicly reported. |
| 2 Finally, I feel somehow unconvinced how Heatmap Visualization could be stated as the contribution of the study. Indeed, with the aid of the heatmap, it’s easier for the viewers to understand your whole results in a glance. However, since your heatmap is just static, there is limited information / usefulness of the heatmap. On the other hand, if you could provide a signaling method using dynamic heatmap and the heatmap-aided dynamic VMA rules is therefore beat the single / static VMA rules, then I would think a more appropriate contribution. | Thank you so much for your brilliant suggestion. We agree that if we can provide a signaling method that uses dynamic heatmaps and heatmap-aided dynamic VMA rules, it will be much better and beat the single / static VMA rules, adding to the existing literature.  However, there are numerous concerns about developing dynamic VMA trading rules (i.e., how to change another heatmap as what signal is emitted; whether investors should change diverse heatmaps as diverse signals emitted; how to design these diverse heatmaps with persuading arguments; providing solid reasons for persuading readers why we should change diverse heatmaps, etc.)  We appreciate your insightful and constructive feedback, which has contributed to the enrichment and broadening of our understanding of our research. However, due to my limited knowledge, we may not be able to resolve the aforementioned concerns promptly with convincing arguments. We will then consider these for future research. (please see P. 23 – P. 24) |
| **Minor suggestions:** |  |
| 1. It’s not clear to me how you construct weekly WMA rules. Since the data is based on daily frequency, do you enter the trade on Mondays only and exit on Fridays only? | Thank you for your valuable comment. Similar to deriving daily data (i.e., (the close daily price at day t / close daily price at day t-1) - 1), weekly data are derived from the same formula (i.e., (the close weekly price at week t / close weekly price at week t-1) -1). Many thanks. |
| 2. Inappropriate citations:  “As a result of inflationary concerns, many people may be motivated to invest in stock markets rather than banks, attempting to understand the stock market, predict stock prices, generate satisfactory profits, and become successful investors” (p. 3) and you cited Roundy et al. (2017). I quickly read the paper and I don’t think this citation is relevant to your statement here, as Roundy et al. (2017) is to provide difference between impact investment and social responsibility investment, I don’t see how this is related to inflation-driven investment.  Another example of inappropriate citation is “according to the efficient market hypothesis (EMH), shock (PS, this should be a typo, it should be stock) prices have reflected all available information” (p. 3), and you cite several studies by Fama.  It’s true that Fama pioneers the conceptualization of efcient fnancial markets, but it’s well known that there are diﬀerent levels of efcient markets, and a fnancial market can be still efcient even some information is not yet reﬂected. So the statement here could be misleading | Thank you for your treasured comment. After carefully reading the study of Rouny e al (2017), we agree with you that this paper is not related to inflation-driven investment. We thus delete this citation in the revised version. Thank you so much. (Please see P. 3)  Thank you for your comment. We correct our typo by using stock prices instead of shock prices in P. 3. Thank a lot. (Please see P. 3)  Thank you for your insightful comment. In fact, there are three types of market efficiency: weak, semi-strong, and strong. That is, past price and volume information is fully reflected in current prices in the weak form; all publicly available information, including insider information, is fully reflected in current prices in the semi-strong form; and all information, including insider information, is fully reflected in current prices in the strong form. We then revise the inappropriate presentation in the previous version. Thank you so much. (please see P. 3) |
| I would like to thank the authors for giving me the opportunity to review your paper. It’s an interesting one but I would think the credibility of the results require much further  investigation.  ***Reference:***  [1] Roundy, P., Holzhauer, H. and Dai, Y., 2017. Finance or philanthropy? Exploring the motivations and criteria of impact investors. *Social Responsibility Journal.*  [2] Fama, E.F., 1970. Efcient capital markets: A review of theory and empirical work. *Journal of Finance*, 25(2), pp.383-417.  [3] Fama, E.F., 1991. Efcient capital markets: II. *Journal of fnance*, 46(5), pp.1575-1617 | Thank you for taking the time to review our paper and for your treasured feedback. We appreciate your interest in our research and understand your concern regarding the credibility of the results. We agree that further investigation is always beneficial. Therefore, we are committed to revising this paper, with the aspiration that our revised version would contribute to the literature and provide insights that can help investors improve their trading strategies. We appreciate your feedback and gratitude for giving us the opportunity to enhance our work. |
| We greatly appreciate your effort in reviewing this paper and your treasured comments provided for our paper, all of which would indeed help us to learn how to write a good academic paper and how to enhance our manuscript. Although we have to make efforts to revise this paper, we indeed learn a lot. We are really appreciated your valuable comments and suggestions. Thank you so much. | |

**REVIEWER 3**

| **Suggestions/comments from the Reviewer** | **Response from the Author(s)** |
| --- | --- |
| There are major and minor concerns relating to the interpretations and the contributions. Please find my detailed concerns in the attached report. | Thank you for your valuable feedback on our study. We greatly appreciate the time and effort you have taken to review our manuscript. Your comments and suggestions will be very helpful in further improving the quality of our work. We are fully committed to making the necessary revisions to our manuscript and addressing the points you have raised. Your insights will play a critical role in helping us to enhance this paper. Thank you so much. |
| **Comments:** |  |
| 1. The author should provide more academic contribution of this research in the abstract. | Thank you for your valuable feedback. In response to your comment, we have revised the abstract to provide the academic contribution of this research in the abstract. Thank you so much. (Please see the abstract section) |
| 2. The authors should discuss more details of the data processing results. | Based on your insightful comments, we will include more details and explanations of the data processing results shown in Tables 4-5 and Figures 2-3 in our study. Thank you very much. (Please see P. 16) |
| 3. The discussion and conclusion should be written separately so the authors can have many space for discussion. | Thank you for your feedback on our manuscript. We appreciate your suggestion to separate the discussion and conclusion sections in order to provide more space for discussion. Thank you again for your valuable insights. (Please see P. 19 – P. 20) |
| We greatly appreciate your effort in reviewing this paper and your treasured comments provided for our paper, all of which would indeed help us to learn how to write a good academic paper and how to enhance our manuscript. Although we have to make efforts to revise this paper, we indeed learn a lot. We are really appreciated your valuable comments and suggestions. Thank you so much. | |
