# Supplementary material for: Using Heatmap Visualization to assess the performance of the DJ30 and NASDAQ100 Indices under diverse VMA trading rules
Source: PLoS One. 2023 May 11;18(5):e0284918. doi: 10.1371/journal.pone.0284918 (PMC10174563; doi:10.1371/journal.pone.0284918)
Supplement: Attachment — Submitted filename: Referee report.docx [file pone.0284918.s001.docx]

**Referee report**

**Manuscript number:** PONE-D-22-31748

**Manuscript title:** Using Heatmap Visualization to assess the performance of the DJ30 and NASDAQ100 Indices under diverse VMA trading rules

**Summary**

This study explores whether using various VMA trading rules would improve investment performance due to the flexibility of VMA trading rules and the aid of heat-map visualization. The results of the empirical analysis show that the NASDAQ100 index outperforms the DJ30 index and that weekly data outperforms daily data when measured by annualized return. In addition, it provided several insights for policymakers and investors in the stock market. Generally speaking, the writing of the manuscript reflects the professionalism of the authors. But before it can be accepted for publication, the authors need to make the following major revisions to the existing manuscript.

**Comments:**

1. The authors mentioned in the introduction part that “Profitability trading using technical trading rules appears to be welcomed by market participants and even academic researchers, which would be the research background and motivation for this study.” It is suggested that the authors restate the motivation in this paper. It could be an unsolved problem from previous studies or a practical problem in the rules of market trading (relevant to the theme of this paper).
2. The fourth point of the study's contribution is not obvious. It is suggested that the authors connect the fourth contribution with the third point (relevant to the heat-map visualization).
3. Some literature about the implications as well as techniques need to be updated carefully:

[a] Leviton, L. C., & Trujillo, M. D. (2017). Interaction of theory and practice to assess external validity. *Evaluation Review*, 41(5), 436-471.

[b] Kwok, J.K.H. (2020), "Does switching trading venues create value? Evidence from Hong Kong", *Journal of Asian Business and Economic Studies*, Vol. 27 No. 2, pp. 209-222. https://doi.org/10.1108/JABES-09-2019-0080

4. It is recommended that the authors add robustness tests following the main empirical work to support the results of the study. For example, the authors mentioned that the DJ30 and NASDAQ 100 indices were chosen because these stock indices enjoy high attention from investors and have high trading volume. Can other similar stock indices be used for comparison to illustrate the effectiveness of the VMA trading rules?

4. It is suggested that the authors redesign **Tables 4** and **Table 5**, as the current version does not meet official publication standards and is detrimental to the reader's understanding.

5. It is suggested that the authors remove the statement to the contribution of this study from the conclusion from **page 19 to** **page 20**. The current version of the conclusion is too redundant. This would give the reader the impression that the results of this study is weak to support the conclusions.

6. The existing reference may have some problems with the format. For instance, the authors mentioned “According to Brock et al. [21] (1992)” on **page 4.** It is suggested that the authors double-check the existing format of reference and revise them carefully in the whole text.

7. It is recommended that the authors recheck the formulas and their interpretation in the research design. The current version seems to be not in good order (e.g. position of formula serial numbers and line spacing).
